# Measles Among the Foreign-Born Population Residing in Spain, 2014–2022: Missed Opportunities for Vaccination

**DOI:** 10.3390/vaccines12121452

**Published:** 2024-12-23

**Authors:** Noemí López-Perea, Teresa López-Cuadrado, Aurora Fernández-García, Juan E. Echevarría, Josefa Masa-Calles

**Affiliations:** 1 National Centre for Epidemiology, Institute of Health Carlos III, 28029 Madrid, Spain; 2 Spanish Consortium for Research in Epidemiology and Public Health (CIBERESP), Instituto de Salud Carlos III, 28029 Madrid, Spain; 3 Department of Preventive Medicine and Public Health, School of Medicine, Autonomous University of Madrid, 28029 Madrid, Spain; 4 National Centre for Microbiology, Institute of Health Carlos III, 28220 Madrid, Spain

**Keywords:** measles, elimination, migrants, Spain

## Abstract

Background/Objectives: Spain has been in a measles elimination phase since 2014. No evidence exists about the distribution of measles cases among the population born outside Spain. The aim of this study was thus to describe the epidemiological situation of measles, stratified by place of birth, during the post-elimination period in Spain. Methods: This is a retrospective study of confirmed measles cases reported to RENAVE between 2014 and 2022. A descriptive analysis of case characteristics (sex, age group, vaccination status, imported case) was performed, was well as an analysis of temporal trends and geographic distribution in measles incidence rate (IR; cases/million inhabitants). All analyses were stratified by place of origin (Spain born vs. born outside Spain). We then performed a sensitivity analysis of those born outside Spain, with the representation of Kaplan–Meier curves taking into account the year of arrival in the country until the onset of measles. Results: Between 2014 and 2022, 951 measles cases were reported in Spain (overall IR: 2.3). Among these, 18.6% (177 cases, IR: 3.0) were born outside Spain. The IRs show differences (*p* < 0.001) in terms of distribution by age group and origin. By age group, children under 5 years had the highest IR, but adults aged 30 years and older reported the highest proportion of cases. The incidence rate ratio (IRR) was 5-fold higher among foreign-born children under 5 years than among native-born children. The measles time trend shows the highest peak in 2019 for foreign-born and native-born (IR: 8.6 and 5.4, respectively), consistent with the European-wide scenario, while only one case of measles was reported in 2022. Geographical variability in incidence rates by region was observed: Catalonia and the Valencian Community accumulated the highest proportion of cases throughout the study period. Among those born outside Spain, the median time from arrival to onset of rash was 6 years. Conclusions: The incidence of measles is 40% higher in Spain’s foreign-born population than in its native-born population. Taking into account the increasing migrant population in Spain, we consider that public health efforts need to be directed towards susceptible groups of people. In this context of advanced elimination, specific interventions for identifying and attending the most vulnerable populations should be designed and implemented.

## 1. Introduction

Its high contagiousness and potential associated complications make measles a disease with one of the highest morbidity and mortality burdens. Despite there being a safe and effective vaccine, measles remains a challenge for health systems and the health of populations.

In 1988, the World Health Assembly proposed a 90% reduction in measles cases and 95% of associated deaths [1] by 2000. Progress in measles elimination differs across World Health Organization (WHO) regions, determined in all cases by the integrity of populations’ immunity to measles. The region of the Americas was the first to reach the goal of measles elimination in 2002 [2,3].

In 1999, at the request of WHO-Europe, the European Advisory Group on Immunization published the Strategic Framework for Measles Elimination in the European Region [4,5] The strategic lines for the goal of elimination are to maintain high vaccine coverage (≥95% with two doses of measles, mumps and rubella vaccine (MMR)), increase vaccination opportunities for potentially susceptible populations and groups, improve epidemiologic surveillance and laboratory investigation of cases and outbreaks, and ensure rapid control of outbreaks. A further recommendation is to improve the capability of healthcare workers to inform the public about the benefits/risks of measles vaccination, with the aim of strengthening demand and improving confidence in vaccination.

In 2019, measles challenged global health systems because of its alarming resurgence. Only the COVID-19 pandemic radically halted that upward trend. In 2022, however, about 136,000 people died of measles worldwide, 43% more than in 2021, most of whom were under the age of 5 years and living in developing countries [6]. At the beginning of 2024, the European Center for Disease Prevention and Control published a report with a threat assessment of the rapid increase in the reporting of measles cases and outbreaks in 2023 in the European Union/European Economic Area [7].

Spain, having achieved 95% coverage with one MMR dose and incidence of less than one case per 100,000 inhabitants, added its name to the list of countries that reached the goal of measles elimination by 2000 [8]. In 2021, the Strategic Plan for Measles and Rubella Elimination in Spain 2021–2025 was updated, adapting to the epidemiological reality and including the new laboratory algorithms proposed by the WHO [9].

The coverage with two doses of MMR in the European Region was 91% in 2023 (95% with one dose) [7]. However, there are significant differences among countries within the region [10].

The report of the last meeting of the Commission for Verification of the Elimination of Measles and Rubella in the European Region, based on data for 2022, concluded that the situation was heterogeneous. Of the 53 countries in the region, measles elimination status could be certified in 33; and while the disease remained endemic in 9 countries, including Romania, Ukraine and Poland, endemic transmission had only been halted in others, such as France, Italy and Germany, in the previous 12 months [11]. In Europe, there are countries in the elimination phase that have traditionally been immigration hosts, like the United Kingdom or Sweden. Pockets of susceptible people, such as adult immigrants who are often not offered vaccination on arrival [12] could pose a challenge to measles control. For this case, RCV recommended supplementary immunization activity (SIA) campaigns for improving vaccination coverages. Measles elimination status is not final, so that if circulation renews, as in Uzbekistan, measles circulation status can be reinstated.

In Spain, MMR was included in the childhood vaccination schedule in 1981, and the second dose was incorporated in 1996. The current MMR schedule envisages administration at age 12 months and again at 3–4 years. In 2019, there was an essential shift from the Child Vaccination Calendar to the Lifelong Vaccination Calendar, in which vaccination with two doses of MMR is recommended for every person born in Spain since 1978 without documented vaccination, by taking advantage of any contact with health services [13].

The fact that there has been no endemic circulation of measles in this country since 2014, the so-called post-elimination phase, meant that the WHO European Regional Verification Committee was able to certify the measles elimination status in Spain in 2017. This post-elimination period is characterized by imported cases of measles coming from other countries where the virus is still circulating, and where there are outbreaks; these are usually small or medium-sized [14].

The migrant population resident in Spain comes from countries where different vaccination policies have been implemented, and this determines the likelihood of a person being immune or susceptible to measles: whereas younger people may have benefited from the scheduled inclusion of MMR, older people may have gone through measles naturally.

The WHO noted that migrant populations may experience more difficulties in accessing health systems and receiving vaccination than populations born in the countries of arrival [15]. In response to this need, it therefore, published a Global Action Plan for Health Promotion among Migrants and Refugees [16], which has been extended until 2030 [17].

There is no scientific evidence at a national level on the distribution and characteristics of measles cases among the population born outside Spain. The aim of this study was thus to describe the epidemiological situation (temporal and geographical) of measles, stratified by place of birth, during the post-elimination period in Spain.

## 2. Materials and Methods

We conducted a retrospective study of confirmed cases of measles (by laboratory, epidemiological link clinical diagnosis) reported to Spain’s National Epidemiological Surveillance Network (Red Nacional de Vigilancia Epidemiológica/RENAVE) across the period 2014–2022. The RENAVE is an essential system for the detection and control of communicable diseases and other health risks, enabling a rapid and coordinated response at a national level [18]. Measles is a notifiable disease in Spain and its enhanced surveillance is one of the cornerstones for maintaining post-elimination status, as outlined in the Measles Elimination Strategic Plan [9]. This ensures the completeness and quality of data for each suspected measles case entering the system.

The population figures published by the National Statistics Institute (Instituto Nacional de Estadística/INE) on 1 January of each year of study [19] were used for the calculation of rates.

We performed a descriptive analysis of the sociodemographic, risk characteristics and place of acquisition of infection of cases by the following factors: sex; age group (under 5 years, 5 through 29 years, and 30 years and older); Autonomous Region (Comunidad Autónoma) of notification; imported case (yes/no); place of origin (born in Spain vs. born abroad); and vaccination status (yes/no), with ‘vaccinated person’ being defined as anyone who had received at least one MMR dose.

In the case of those born outside Spain, the continent and main countries of origin were described. The association between place of origin and the mentioned qualitative covariates was explored using Pearson’s chi-squared test. Measles incidence rates (cases/million population) were calculated by age group and year, along with the incidence rate ratio and its corresponding confidence interval (IRR, 95% CI).

Time trends in measles incidence rates and the geographical distribution of rates and proportions were plotted. A sensitivity analysis was performed for the migrant population group, using Kaplan–Meier curves, with follow-up time being defined as time (in years) from arrival in the country to the onset of measles, indicating the median time.

We performed the analysis using Stata^©^ v17 (StataCorp. 2021. Stata Statistical Software: Release 17. College Station, TX, USA: StataCorp LLC.)

All results were reported by place of origin (born in Spain vs. born abroad), excluding all cases of measles among the migrant population not residing in Spain (40 cases (4.0%)).

## 3. Results

From 2014 through 2022, 951 cases of measles were reported among the population living in Spain; of these, 18.6% (177) had been born outside Spain. Depending on their place of origin, differences were found between cases with known vaccination status (91.8%), with the unvaccinated proportion being higher among residents born outside Spain than among Spanish natives (67.3% vs. 62.8%). A similar situation was observed in the proportion of imported cases (23.6% vs. 6.2%) (Table 1).

The overall measles incidence rate across the study period was 2.3 cases per million population, with incidence being higher among those born outside than among those born in Spain (incidence rate (IR): 3.0 vs. 2.1; IRR = 1.4 (95% CI: 1.2–1.6)).

By age group, the highest proportion of cases was reported among adults aged 30 years and over (49.7%), and the highest incidence occurred among children under 5 years of age (IR: 9.1 cases/million). After stratification by place of origin, rates in children under 5 years of age were significantly higher among those born abroad than among those born in Spain (IR: 42.3 vs. 8.2; IRR: 5.1; 95% CI (3.0–8.3)) (Table 2). In the 5–29 age group, rates were likewise significantly higher among residents born in other countries (IR: 5.4 vs. 2.4; IRR: 2.2; 95% CI: (1.7–2.9)), whereas in the 30-and-over age range, the IRRs were similar between the two groups (Table 2).

**Figure 1:**
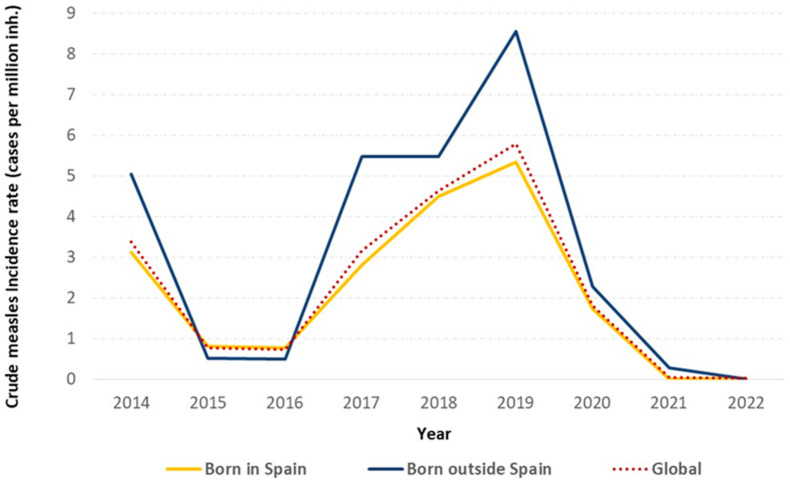
Crude measles incidence rate by birth origin and year. Spain, 2014–2022.

The measles time trend shows the highest peak in 2019 for both groups (IR: 8.6 and 5.4, respectively), while only one case of measles was reported in 2022 (Figure 1).

**Figure 2:**
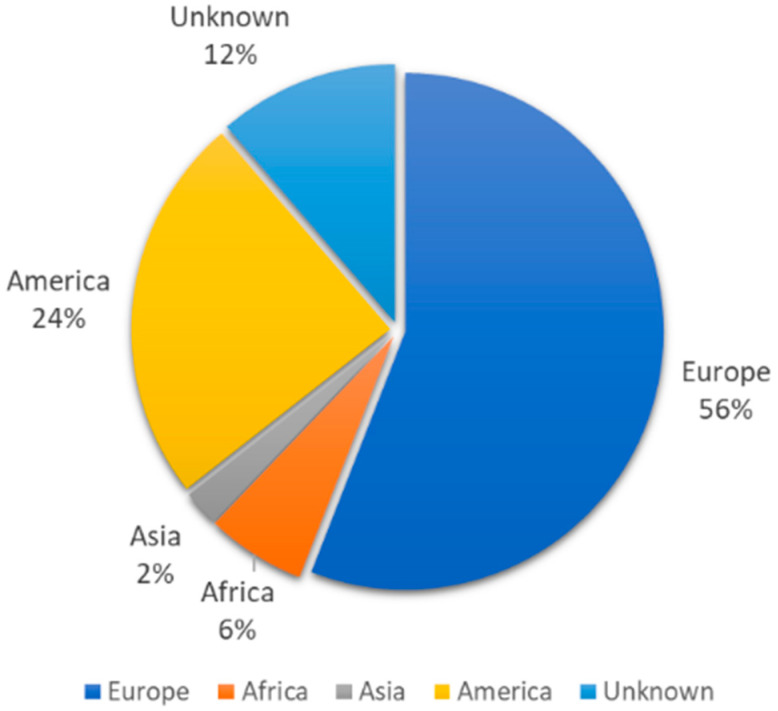
Measles cases born outside Spain, by continent. Spain, 2014–2022.

Of the 177 cases reporting a foreign origin, 55.9% came from European countries, with Romania (32 cases), Italy (23 cases) and Ukraine (18 cases) being the most frequent countries of origin. The next most frequently sourced continents were the Americas (24.3%), Africa (6.2%) and Asia (2.3%) (Figure 2).

**Figure 3:**
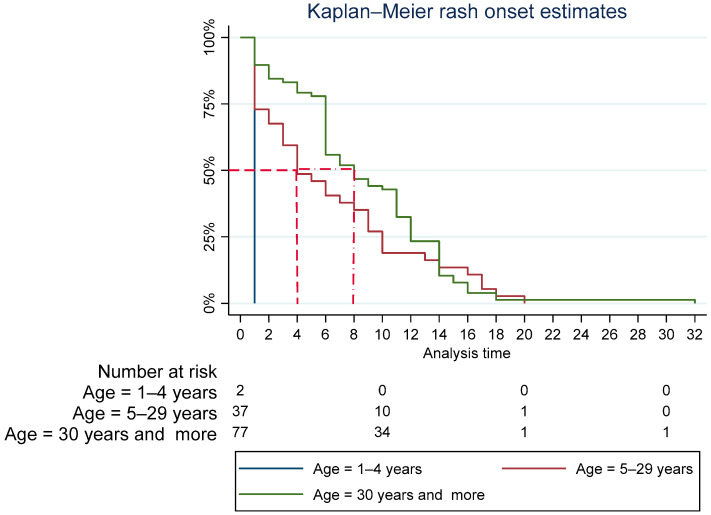
Kaplan Meier curves for measles cases born outside Spain. 2014–2022.

Among those born outside Spain, the median time from arrival to onset of rash was 6 years. A breakdown by age group showed that the median time was 1 year, 4 years and 8 years for those aged under 5 years, 5–29 years, and ≥30 years, respectively (Figure 3).

Wide geographic variability was observed in measles reporting by Autonomous Region. The regions with the most cases of measles were Catalonia (43.2%) and Valencia (19.6%), accounting for 63% of all notifications across Spain, followed by Madrid (8.1%), Castile-La Mancha (7.3%), and Andalusia (5.6%) (Table S1). The percentage of cases of foreign origin in a given region varied from 30.2% in Andalusia to 10.1% in Castile-La Mancha, with Catalonia and Valencia registering the highest incidences among the population of foreign origin (IR: 6.3 and 4.2, respectively) (Table S1 and Figure S1).

## 4. Discussion

In the post-elimination phase in Spain, with endemic transmission of the measles virus interrupted, the risk of an imported case generating an outbreak depends on the susceptibility of the population group to which it belongs, and/or the area in which the imported case arrives. This rate of susceptibility may depend on, among other things, vaccination policies and coverage in the countries where people come from.

The years prior to the COVID-19 pandemic (2018–2019) saw an alarming resurgence of measles cases and outbreaks in all WHO regions, due to uneven or sub-optimal vaccination coverage (<95% with two doses of MMR) [3,20].

Following the sharp global drop in measles reporting due to pandemic barriers, lockdown, and distancing measures (2020 and 2021), there has been a new resurgence of the disease in the last two years [6,21]. The pandemic also had an impact on health systems in terms of the performance of immunization programs, as in the case of the European Region, where MMR coverage decreased from 2020 through 2022 (falling from 96% to 93% with one MMR dose between 2019 and 2022, and from 92% to 91% with two doses) [3,22]. This, coupled with the recovery of the international movement of people, resulted in more than 60,000 cases of measles and 13 related deaths being reported in the region in 2023, and the scenario has become even worse in 2024, with more than 95,000 cases having been reported to date [21,23].

In Spain, the COVID-19 pandemic impacted measles epidemiology in much the same way as it did in neighboring countries, with a drastic reduction in case reports since the first lockdown, as well as in 2021 (two cases) and 2022 (one case). For vaccination coverage, first-dose data, which had traditionally remained above 95% at both national and regional levels since 2000, decreased in some regions to below 90% in 2021, but remained above 95% at a national level [14]. Vaccination coverage with two doses, which traditionally ranged around 90%, was even more affected. However, thanks to the uptake and vaccination efforts made by the Autonomous Regions, vaccination coverage in 2022 was similar to the rates recorded prior to the pandemic [24,25].

In the current epidemiologic situation here in Spain, the probability of exposure to the measles virus is extremely random. A susceptible person can spend years, or even a lifetime, without being exposed to the virus, especially if he/she lives in rural areas and travels little. The reported cases are, therefore, neither representative of nor proportional to the susceptible population, as the opportunity for exposure depends on other factors, such as population density, intra-group population dynamics, or the heterogeneity of the origin of the population. Reported measles cases are, therefore, a ‘raw’ indicator of the level of susceptibility of the population group to which the case belongs. The WHO estimates that if the level of susceptibility in a population is above 5%, there is a risk of measles spread and a threat to the elimination status [26].

In our study, the proportion of measles cases in foreign-born subjects accounted for 18.6% of all reported cases, while in the same period of time, the percentage of foreign-born people in the general population was 13.8%. The foreign population resident in Spain increased by 25.5% (more than 1.5 million people), rising from 12.8% in 2014 to 15.7% in 2022 [19]. In 2022, Spain registered one of the highest entry rates into the European Region in terms of foreigners per 100,000 inhabitants, and ranks fourth globally in the reception of permanent immigrants [27].

The age distribution of cases shows differences when stratified by place of origin. High rates in children under 5 years of age born abroad are explained both by the low denominator and by vaccination schedules in the countries of origin, where the second dose of MMR is usually given from the age of 5 onwards [28]. The group of migrants aged 5–29 years has an incidence rate which is double that of people of this age born in Spain, and is thus a vulnerable group that requires special attention when it comes to measles.

Studies have shown that the immune status related to measles among migrants in Europe is worse than that of the native resident population, mainly because they have not received the doses or potential MMR boosters in their home countries [29]. In our study, the proportion of imported cases was significantly higher among people born outside Spain (23.6% vs. 6.1%), which can be explained by the fact that it is common among the resident foreign population to travel to their countries of origin, or to interact with family or friends coming from those countries, so that the risk of exposure to the virus, if it still continues to circulate in their country, is higher.

On the other hand, there is evidence of increased vaccine hesitancy among migrants, partly because xenophobic feelings of the native population reduce confidence in the health system of the country of arrival, thus intensifying anti-vaccination sentiments among migrants [30], which may in turn lead to outbreaks of immune-preventable diseases, even in places with high vaccination coverages [31].

The WHO Global Action Plan to Promote the Health of Refugees and Migrants, 2019–2023, flags the potential for the host countries’ own legal systems to be a source of inequities in access to health resources for these vulnerable groups [32]. It is, therefore, necessary to engage in self-criticism and assess any potential impediments barring access to health resources as well as the timeliness and appropriateness of the health information provided to these population groups.

For some Spanish geographic areas, it should be borne in mind that there are population groups with anti-vaccination feelings fed by people from European countries where these movements are common, such as in the Canary Islands and Balearic Isles [33].

As far as the source continents are concerned, while more than 50% of the migrant population during the study period came from Latin America, barely 25% of measles cases were born there. In contrast, 55.9% of all measles cases reported across the study period and born abroad were of European origin. The countries with the highest number of cases were Romania, Italy and Ukraine. In Romania and Ukraine, measles has been endemic, and they have experienced major epidemics for several years with overwhelming public health consequences.

In Romania, MMR is given at ages 12 months and 5 years [28]. Since 2014, vaccination coverage with one MMR dose has barely reached 89%, falling to 83% in 2022 [10]. Susceptible pockets have been reflected in the explosion of cases reported between 2016 and 2020. In 2023, more than half of the cases were under 5 years old, mostly unvaccinated [34]. That year, the Romanian community in Spain accounted for 1.1% of the total population, with around 540,000 inhabitants [19].

Consideration should be given to the possibility that some of the migrants of Romanian origin also present the added challenge of belonging to the Roma community. The possible condition of associated marginality could not only render it more difficult for the health system to identify them, but also make for poor access to healthcare services [35]. This might better explain their lower vaccination coverage vis-à-vis native populations, than would refusal of vaccination due to anti-vaccination feelings [30].

Italy has included MMR administration at ages 12 months and 5/6 years in its vaccination schedule [28], and in 2023, was able to prove that measles virus circulation had been interrupted for 12 months [11]. However, from 2017 to 2020 it experienced major outbreaks, with about 8500 cases. While first-dose vaccination coverage has never actually reached 95%, with values remaining closer to 90%, since 2021, this target figure has been approached [10].

The flow of people from Ukraine to Spain has greatly increased since the beginning of the conflict (rising from an inward-bound figure of 9836 in 2021 to one of 85,978 in 2022), with the under-15 age group accounting for around 33% of all arrivals in 2022 [36]. Although Ukraine administers MMR to children at the ages of 12 months and 6 years [28], vaccination coverage was already below 80% before the war with Russia (in 2016, it fell to 42%) [37]. Political instability, as well as mass and social media-backed efforts to undermine the health system, and vaccination campaigns in particular, led to an increase in anti-vaccination feelings in the country [37]. This, coupled with the difficulties in accessing health services resulting from the war that began in 2022, keeps the country in a difficult situation regarding immunity to immune-preventable diseases, such as measles. The direct consequence of the decline in population immunity was an exponential increase in cases, outbreaks and even deaths during the 2018–2019 measles epidemic, with more than 110,000 cases and 40 associated deaths [37,38].

Since 2019, both the Common Immunization Schedule throughout life [13] and the Accelerated Vaccination Schedule [39], which is applied in situations such as those with a migrant or displaced population, have been published annually. In Spain, the healthcare competencies are transferred to the autonomous communities, which are responsible for identifying vulnerable population groups. Vaccination of immigrants is included in primary care programs, and any contact with the health system is used as an opportunity to update vaccinations (e.g., pertussis vaccination in pregnant women or MMR vaccination after childbirth). For the specific case of Ukrainian war refugees, an Action Guide was issued in 2022 in response to the arrival of displaced persons from Ukraine [40], setting out this population’s vulnerable status and the measures to be taken in terms of vaccination, all of which were detailed in a specific document [41]. Our results suggest that, in general, the resident population of foreign origin has a worse vaccination status than that born in Spain. However, one possible bias could be the underreporting of vaccination status among migrants, which is higher than among Spain-born cases (15.7% vs. 10.6%; *p* = 0.022) (Table 1).

The results of the second seroprevalence study, based on data from 2017–2018 [42], are consistent with the distribution of measles cases in the current phase. In terms of immune status with respect to measles, people who were born from 1988 to1997, and at the time of data-collection were 20 to 29 years old, had an antibody titer level of below 87%. These are the traditionally susceptible cohorts, meaning that they did not have the opportunity to receive the vaccine because of the low initial coverage after its inclusion in the calendar, and were at the same time less likely to develop natural antibodies due to the decline in the incidence of the disease, limiting their possibility of exposure to the wild virus [24]. It was noted that there were greater differences between those born in and outside Spain, in cohorts born between 1988 and 1997, among whom the percentage of seropositivity for measles is lower. These differences are not significant, however, and in addition, the sample was too small for disaggregated age group calculations [42].

Anti-vaccination feelings or vaccination hesitancy in the country of origin accompany migrants or displaced persons to the destination country and pose an additional challenge to health systems and host professionals. At the 65th World Health Assembly in 2022, a report was presented by the Behavioral Sciences for Better Health Initiative [43], with the aim of promoting the systematic use of behavioral and social sciences in public health. This initiative aims to broaden the health approach by improving understanding of people’s health behaviors, so as to be able to design more effective interventions. This approach from the social and behavioral sciences needs to be incorporated as an essential element in public health interventions in general, and in health vulnerable groups in particular. The case of the displaced Ukrainian population is an example of where such an approach could be applied.

The assumption that the resident population of foreign origin generally has a worse immune status than that of the host country [44], especially if the latter is in the post-elimination phase, should not only be made with caution, but should also take into account the vaccination coverage of the country of origin and its elimination status. In Spain, most of the resident migrant population comes from Latin America, but this does not translate into a proportional number of cases, due to the virus’ limited circulation in that region for a good number of years (elimination of measles having been declared for that region in 2002). Nonetheless, it is true that countries such as Colombia, the country of origin of the majority of cases of measles of American origin in this study (10; 5.6%), has barely achieved 95% coverage with one MMR dose since 2014 (in 2018 and again in 2019), falling below this figure even before the pandemic [45].

Catalonia and the Autonomous Region of Valencia are the regions with the highest percentage of total cases and cases reported in people born outside Spain during the study period. This could be due to the wider variety of origins of the immigrant population in these regions, to the increased transit of international flights, or to different population dynamics still to be examined.

During the post-elimination period, it takes an average of 6 years from arrival in Spain for the resident population of foreign origin to become infected with measles (4 years, if subjects are in the 5–29 age group). In the case of school-age children, the active work of the health system in recruiting vaccine candidates is of particular importance.

Spain is increasingly a country of reception of migrant populations, with the potential risk of generating health inequities. The system needs to undergo a review to assess whether migrants’ potential contacts with the health sector are being sufficiently exploited to offer them vaccination (e.g., when they are about to make an international trip, such as to their country of origin; by bringing children to the pediatrician; or when they make a medical visit for other health reasons) [46]. While this measure is included in the Strategic Plan for Measles Elimination for the general population [9], the particular vulnerability of this population group should be emphasized. In this regard, health professionals are key, both in identifying susceptible individuals and in conducting health education and promoting correct vaccination.

Health personnel should receive continuous and up-to-date training in vaccination schedules and the implementation of accelerated guidelines if needed, and be made aware of the importance of maintaining the measles elimination status here in Spain. Finally, this policy should be complemented by incorporating the views of professionals in behavioral and social sciences, as proposed by the WHO initiative, in order to comprehensively address barriers linked to health behaviors, such as vaccine hesitancy.

## 5. Conclusions

This study shows that during the post-elimination period, the incidence of measles is 40% higher in Spain’s foreign-born population than in its native-born population. In the migrant population, the median time from arrival in Spain to contracting measles is estimated at approximately 6 years. More than 50% of measles cases in the migrant population are of European origin. Lastly, the regions with the highest number of reported measles cases as well as the highest rates of foreign residents are Catalonia and Valencia.

Despite the fact that one of the current challenges facing Spain is the increase in migratory flows, health systems (including epidemiological surveillance and laboratory systems) have managed to maintain the elimination status of the disease.

All efforts must be directed at any potentially susceptible person. The health system is responsible for identifying the most vulnerable groups so that specific intervention measures can be designed, such as reviewing the vaccination schedule of anyone born outside Spain upon arrival or improving the collaboration with migrants’ communities and the health system, which will help mitigate susceptibility to measles and thus maintain the current elimination status.

## Figures and Tables

**Table 1:** Characteristics of measles cases by birth origin. Spain, 2014–2022.

	Total		Born Outside Spain		Born in Spain		*p*-Value
	n	%	n	%	n	%	
**Cases**	951	100	177	18.6	774	81.4	
**Sex**							0.854
Male	483	50.8	91	51.4	392	50.6	
Female	468	49.2	86	48.6	382	49.4	
**Age group**							<0.001
0–4 years	171	18.0	20	11.3	151	19.5	
5–29 years	307	32.3	81	45.8	226	29.2	
≥30 years	473	49.7	76	42.9	397	51.3	
**Imported case**							<0.001
Yes	89	9.4	41	23.7	48	6.2	
No	862	90.6	136	76.8	726	93.9	
**Vaccination status**							0.022
Vaccinated	239	25.1	31	17.1	208	26.6	
Non vaccinated	600	63.1	118	67.3	482	62.8	
Unkown status	112	11.8	28	15.7	84	10.6	

**Table 2:** Crude measles incidence rates and incidence rate ratios by age group and birth origin. Spain. 2014–2022.

Age Group	Total			Born Outside Spain			Born in Spain			IRR **	CI (95%)
	Measles Cases (n)	%	IR * (Per Mill)	Measles Cases (n)	%	IR (Per Mill)	Measles Cases (n)	%	IR (Per Mill)		
**<51** **5 years**	171	18.0	9.1	20	11.7	42.3	151	88.3	8.2	5.1	(3.0–8.2)
**5–29 years**	307	32.3	2.8	81	26.4	5.4	226	73.6	2.4	2.2	(1.7–2.9)
**≥30 years**	473	49.7	1.6	76	16.1	1.8	397	83.9	1.6	0.8	(0.6–1.1)
**Total**	951	100	2.3	177	18.6	3.0	774	81.4	2.1	1.4	(1.2–1.6)

* IR: incidence rate = measles cases (n)/10^6^ inhabitants; ** IRR: incidence rate ratio.

## Data Availability

The data presented in this study are partially available on request from the corresponding author due to the data are collected as part of mandatory surveillance to provide information for the National Measles Elimination Plan. Most of the individualised information is restricted and is only allowed to be handled as aggregated analysis.

## Supplementary Materials

The following supporting information can be downloaded at: https://www.mdpi.com/article/10.3390/vaccines12121452/s1. Table S1: Measles cases and crude incidence rates by birth origin and region, Spain, 2014–2022; Figure S1: Geographical distribution of measles cases (%) by birth origin and region, Spain, 2014–2022.
