# Supplementary material for: Measles Among the Foreign-Born Population Residing in Spain, 2014–2022: Missed Opportunities for Vaccination
Source: Vaccines (Basel). 2024 Dec 23;12(12):1452. doi: 10.3390/vaccines12121452 (PMC11680080; doi:10.3390/vaccines12121452)

Supplementary Material

Table S1. Measles cases and crude incidence rates by birth origin and region. Spain, 2014-2022

Region	Born in Spain			Born outside Spain			Total		
	n	%	IR	n	%	IR	n	%	IR
**Andalucía**	37	3.9	0.5	16	9.0	2.7	53	5.6	0.7
**Aragón**	12	1.3	1.2	1	0.6	0.8	13	1.4	1.1
**Asturias**	1	0.1	0.1	0	0.0	0.0	1	0.1	0.1
**Baleares**	17	1.8	2.2	2	1.1	1.1	19	2.0	2.0
**Canarias**	1	0.1	0.1	5	2.8	2.1	6	0.6	0.3
**Cantabria**	0	0.0	0.0	0	0.0	0.0	0	0.0	0.0
**Castilla y León**	5	0.5	0.2	3	1.7	2.5	8	0.8	0.4
**Castilla -La Mancha**	62	6.5	3.8	7	4.0	4.3	69	7.3	3.8
**Cataluña**	340	35.8	6.1	74	41.8	7.3	414	43.5	6.3
**Valencia**	148	15.6	4.0	36	20.3	5.6	184	19.3	4.2
**Extremadura**	9	0.9	1.0	0	0.0	0.0	9	0.9	0.9
**Galicia**	16	1.7	0.7	2	1.1	2.2	18	1.9	0.8
**Madrid**	56	5.9	1.2	21	11.9	2.7	77	8.1	1.4
**Murcia**	9	0.9	0.8	1	0.6	0.5	10	1.1	0.8
**Navarra**	45	4.7	9.0	7	4.0	12.4	52	5.5	9.4
**País Vasco**	14	1.5	0.8	1	0.6	0.7	15	1.6	0.8
**La Rioja**	2	0.2	0.8	1	0.6	2.9	3	0.3	1.1
**Ceuta**	0	0.0	0.0	0	0.0	0.0	0	0.0	0.0
**Melilla**	0	0.0	0.0	0	0.0	0.0	0	0.0	0.0
**Total**	**774**	**81.4**	**2.1**	**177**	**18.6**	**3.9**	**951**	**100**	**2.3**

Figure S1. Geographical distribution of measles cases (%), by birth origin and region. Spain: 2014-2022.

Measles cases (%).Population born outside Spain. Spain, 2014-2022

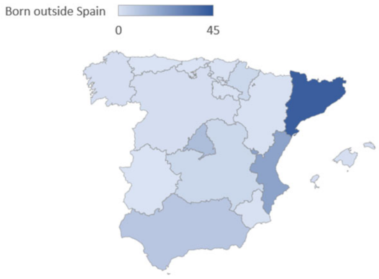

Measles cases (%). Spain-born population. Spain, 2014-2022

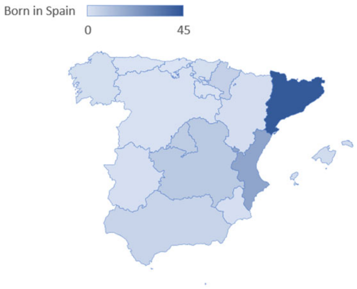

Supplement: Supplementary file 1 [file vaccines-12-01452-s001.zip › vaccines-3345844-supplementary.pdf]
